# Dengue Infection Increases the Locomotor Activity of *Aedes aegypti* Females

**DOI:** 10.1371/journal.pone.0017690

**Published:** 2011-03-08

**Authors:** Tamara N. Lima-Camara, Rafaela V. Bruno, Paula M. Luz, Márcia G. Castro, Ricardo Lourenço-de-Oliveira, Marcos H. F. Sorgine, Alexandre A. Peixoto

**Affiliations:** 1 Laboratório de Biologia Molecular de Insetos, Instituto Oswaldo Cruz, FIOCRUZ, Rio de Janeiro, Brazil; 2 Laboratório de Pesquisa Clínica em DST & AIDS, Instituto de Pesquisa Clínica Evandro Chagas, FIOCRUZ, Rio de Janeiro, Brazil; 3 Laboratório de Transmissores de Hematozoários, Instituto Oswaldo Cruz, FIOCRUZ, Rio de Janeiro, Brazil; 4 Programa de Biologia Molecular e Biotecnologia, Instituto de Bioquímica Médica, Universidade Federal do Rio de Janeiro, Rio de Janeiro, Brazil; 5 Instituto Nacional de Ciência e Tecnologia em Entomologia Molecular (INCT-EM), Rio de Janeiro, Brazil; Agency for Science, Technology and Research - Singapore Immunology Network, Singapore

## Abstract

**Background:**

*Aedes aegypti* is the main vector of the virus causing Dengue fever, a disease that has increased dramatically in importance in recent decades, affecting many tropical and sub-tropical areas of the globe. It is known that viruses and other parasites can potentially alter vector behavior. We investigated whether infection with Dengue virus modifies the behavior of *Aedes aegypti* females with respect to their activity level.

**Methods/Principal Findings:**

We carried out intrathoracic Dengue 2 virus (DENV-2) infections in *Aedes aegypti* females and recorded their locomotor activity behavior. We observed an increase of up to ∼50% in the activity of infected mosquitoes compared to the uninfected controls.

**Conclusions:**

Dengue infection alters mosquito locomotor activity behavior. We speculate that the higher levels of activity observed in infected *Aedes aegypti* females might involve the circadian clock. Further studies are needed to assess whether this behavioral change could have implications for the dynamics of Dengue virus transmission.

## Introduction

Over the last decades, Dengue outbreaks have been a major public health concern in many parts of the World, where Dengue epidemics have been registered with a significant number of deaths [1]. There are four antigenically distinct RNA viruses that can cause the disease, and in Brazil, three Dengue serotypes (DENV-1; DENV-2 and DENV-3) have co-circulated in several areas and caused some severe Dengue epidemics [2].

*Aedes aegypti* (Diptera: Culicidae) is the urban vector of Yellow Fever and Dengue viruses. This diurnal mosquito is very anthropophilic and endophilic, being commonly found in urban and suburban areas, especially where house and human densities are high and where it seems to live longer and disperse to short distances (e.g. [3]–[5]).

It is known that parasites can alter vector behavior (reviewed by [6]–[8]) and a number of studies have reported behavioral changes in *Ae. aegypti* infected with pathogens and symbionts. For example, Rossignol et al [9] observed that *Ae. aegypti* females experimentally infected with an avian malaria parasite, *Plasmodium gallinaceum*, take more time to locate blood in guinea pigs than the uninfected ones. Rowland and Lindsay [10] studied the flight activity of females of this species infected with the filarial parasite *Brugia pahangi* and observed a decrease in flight capacity in heavily-infected mosquitoes under laboratory conditions. Recently, it has been shown that the infection by the symbiotic bacterium *Wolbachia* can also drastically alter this mosquito's behavior and physiology [11]–[14].

In the current study, we investigated whether infection with Dengue virus causes changes in the locomotor activity behavior of *Ae. aegypti* females under laboratory conditions.

## Methods

### Infection of Mosquitoes with the Dengue virus

The Paea strain of *Ae. aegypti* was used in all infection experiments. This laboratory strain is known to be highly susceptible to Dengue virus serotype 2 (DENV-2) infection [15]. Mosquito colony was reared according to procedures described in [16]. Males and females remained together and were fed with 15% sucrose solution since emergence.

Five-day-old female mosquitoes were individually infected by intrathoracic inoculation with 0.21 µl of L-15 (Leibovitz) Medium containing Dengue virus (DENV-2 strain FIOCRUZ-66985 [17]) in a concentration of 10^7^ PFU using a Nanoject microinjector (Drummond Scientific). Control mosquitoes were intrathoracically inoculated with 0.21 µl of only L-15 (Leibovitz) Medium.

To verify the success of the experimental infections, the heads of a number of mosquitoes that were inoculated with virus and that were alive by the end of the locomotor activity experiments (around 8–10 days after inoculation), plus negative controls, were tested for Dengue infection via RT-PCR, as described below. The results indicated that over 95% (70/73) of the mosquitoes inoculated with the Dengue virus were infected (data not shown).

### Detection of Dengue virus in mosquitoes

Mosquito heads were separated from bodies on a metal plate placed on dry ice and viral RNA was extracted from each head using the QIAmp Viral Mini Kit (Qiagen) according to the manufacturer's protocol. RT-PCR for detecting DENV2 was conducted in a PCR System 9700 GeneAmp (Applied Biosystems) using SuperScript™ One-Step RT-PCR with Platinum® *Taq* (Invitrogen) and Dengue virus consensus primers D1 and D2 [18], followed by a semi-nested PCR on the resulting product using Go Taq Green Master Mix (Promega) and primers D1 and TS2 [18]. PCR products were electrophoresed on 2.5% agarose gels. A band of 119 pb corresponding to DENV-2 could be seen under UV light in the infected mosquitoes.

### Analysis of the locomotor activity behavior

The activity of infected and uninfected control *Ae. aegypti* females was recorded using a larger version of the Drosophila Activity Monitor (TriKinetics) as described in [19]. After inoculation with Dengue virus or L-15 medium, females were individually placed in glass tubes (1 cm×7 cm) with a cotton plug soaked in 15% sucrose solution and these tubes placed in the Activity Monitor inside a Precision Scientific Incubator Model 818 under a constant temperature of 25°C and a photoperiod of 12 hours of light and 12 hours of dark (LD 12∶12). For each mosquito, the total locomotor activity of 1 hour-intervals was recorded for about seven days after inoculation. The data of individuals that died during the experiments were excluded, and the data analysis was carried out comparing the activity data of infected and uninfected mosquitoes from the second to sixth day after inoculation.

## Results

Table 1 shows the mean hourly locomotor activity of control and Dengue virus infected females in four different experiments. We observed that infected females of *Ae. aegypti* showed more activity than controls in all experiments. The relative increase in activity ranged from ∼10% to more than 50%. A two-way ANOVA indicated a significant difference in the activity between infected and uninfected control females (p<0.01). Although a significant difference in the overall activity levels was also observed between experiments (p<0.01), the interaction between experiments and infection was not significant (p = 0.82) indicating that the difference between infected and uninfected females was consistent.

**Table 1:** Activity increase in Dengue infected *Aedes aegypti* females.

Experiment	Status	N	Mean activity per hour* (± SEM)	Relative increase of activity in infected mosquitoes (%)
1	Control	17	5.06±1.16(4.73±1.15)	52.6 (48.0)
	Infected	23	7.72±1.27(7.00±1.26)	
2	Control	53	11.22±1.45(10.52±1.49)	30.8 (30.5)
	Infected	74	14.68±1.50(13.73±1.49)	
3	Control	45	9.84±1.19(8.97±1.15)	43.2 (45.3)
	Infected	66	14.09±1.24(13.03±1.23)	(13.03±1.23)	
4	Control	70	11.88±1.01(10.95±1.00)	13.3 (10.3)
	Infected	83	13.46±1.13(12.39±1.06)

*The numbers in parenthesis refer to the activity excluding the light-on/light-off transition.

**Figure 1:**
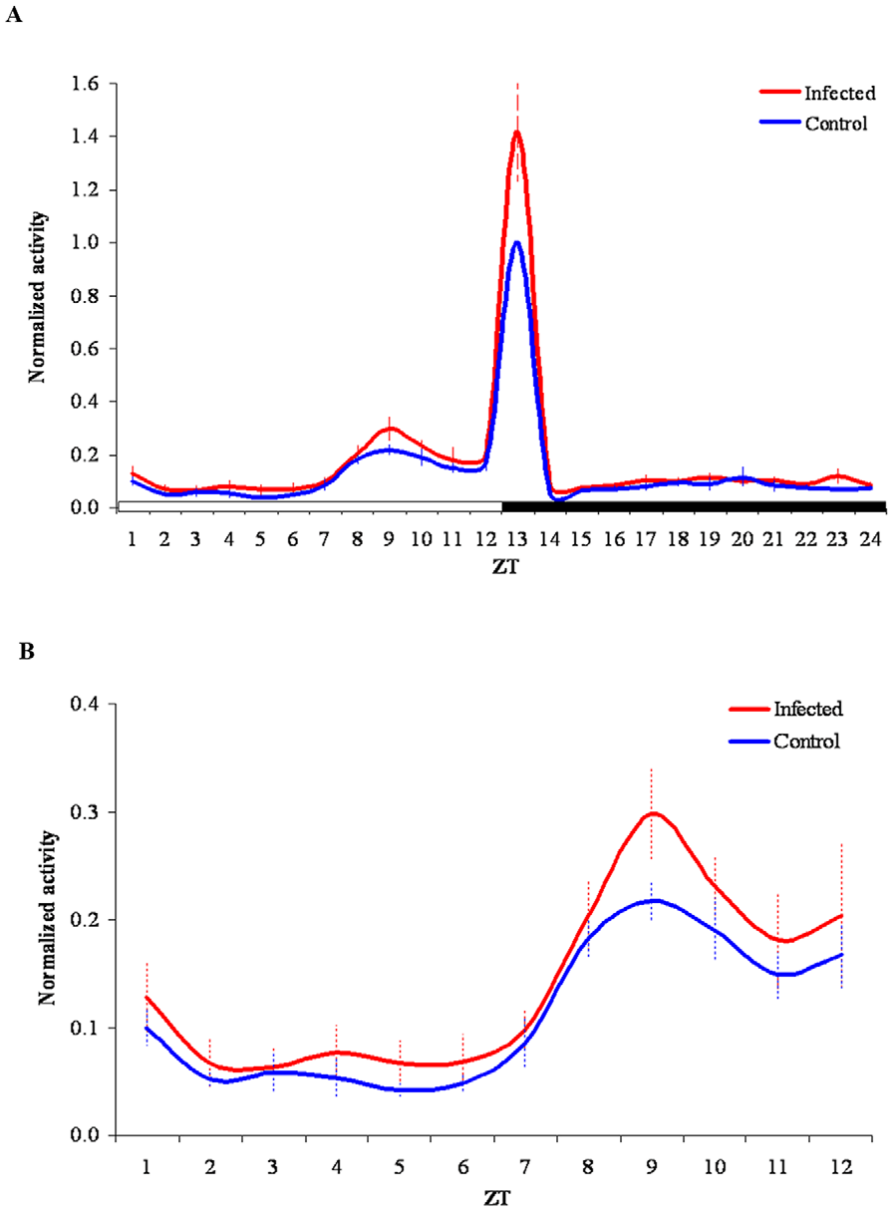
Locomotor activity of control (blue line) and infected (red line) *Ae. aegypti* females under LD 12∶12. Lines represent the hourly mean activity (+/− SEM) of control and infected females in the four experiments, normalized to the peak of activity of each respective control. The grey shadow represents the dark phase. ZT is the Zeitgeber Time. Light turns on at ZT 0 and turns off at ZT 12. Panel (A) shows a full LD cycle while panel (B) shows only the photophase.

Figure 1 shows the normalized locomotor activity patterns of infected and control females during a full LD 12∶12 cycle (Fig. 1A) or during the photophase (Fig. 1B). As previously reported in the literature (reviewed in [20]), *Ae. aegypti* is a diurnal species showing higher activity levels towards the end of the photophase (“late afternoon”) and a characteristic startle response to the abrupt light-on/light-off transition [21]. The comparison of the normalized locomotor activity patterns of infected and uninfected females shows that Dengue infection causes an increase in activity throughout the 24 hour period. Although this effect is most dramatic during the light-on/light-off transition (Fig. 1A), an increase in activity is also seen throughout the day and night in infected females, especially during the “natural” activity peak occurring around ZT 9 and in the last hours of the photophase (Fig. 1B). In fact, this increase in activity associated with Dengue infection is still significant (p = 0.012) even when we exclude the light-on/light-off transition (Table 1). In summary, our results indicate that the locomotor activity of infected females is consistently increased when compared to that of uninfected females.

## Discussion

Very little is known about the effects of viral infection on *Aedes* mosquitoes. Several authors have shown that Dengue virus exhibits a remarkable tropism for the mosquito nervous tissues. Linthicum et al [22] studied the tropism of DENV-3 in parenterally infected female *Aedes aegypti* mosquitoes using immunocytochemical methods and observed that the nervous tissues were among the first tissues to be infected. In fact, these authors suggested that the nervous system is the primary site of virus amplification in mosquitoes infected using this method [22]. Several years later, Salazar et al [23] corroborated these findings by showing that in mosquitoes orally infected with DENV-2, the nervous tissues are among the first to be infected, presenting detectable levels of viral antigens 5 days after an infective blood meal. Interestingly, these authors also showed that heads and salivary glands were the only tissues where viral antigens continued to accumulate throughout the 21 days observed in their study. All other mosquito infected tissues presented a decrease in the infection rate.

This remarkable tropism of Dengue virus for the insect nervous tissues led us to hypothesize that the infection might have some role in modulating the vector locomotor activity behavior, since it is known that activity rhythms in *Drosophila* and other Diptera are regulated by circadian clock neurons in the brain (reviewed in [24],[25]). In fact, our results show that although the daily activity patterns of DENV-2 infected and uninfected mosquitoes are similar, the total level of activity is clearly increased upon infection. This increase is most evident in the light-on/light-off transition (Fig. 1A), an observation that is particularly interesting considering that the visual system is also highly infected [22],[23]. However, it is important to mention that this effect is also clearly detected in the “natural” activity peak occurring during the last hours of the photophase (Fig. 1B), which is under circadian control [20],[21], indicating that a similar effect is likely to occur in nature.

Other authors have already observed alterations in *Aedes* behavior induced by virus infection. Grimstad et al [26] studied the feeding behavior of *Ae. triseriatus* females infected with La Crosse virus and reported that infected mosquitoes tend to probe more and engorge less than uninfected females. These results are in accordance with those obtained by Platt et al [27], who showed that the time required for feeding by DENV-3 infected mosquitoes was significantly longer than that required by uninfected mosquitoes. In contrast, Putnam and Scott [28] observed that DENV-2 infection did not alter *Ae. aegypti* female blood-feeding duration and efficiency in an uninfected host. An explanation for this difference might be that these authors infected mosquitoes with different Dengue virus (3 and 2, respectively) and that Putnam and Scott [28] fed mosquitoes 14 days after an intrathoracic infection while Platt et al [27] only observed significant differences in mosquitoes fed 5, 8 and 11 days after infection. In our study, we observed locomotor activity differences in DENV-2 infected mosquitoes 2 to 6 days after intrathoracic infection.

A considerable amount of information is currently available on the *Aedes aegypti* immune response to Dengue virus infection [29]–[31]. Several authors have shown an association between circadian rhythms and infection/immunity in insects (e.g. [32]–[34]). For example, Shirasu-Hiza et al [32] showed that *Drosophila* infected with bacterium exhibit disrupted circadian activity rhythms and that clock gene mutants are more susceptible to infection than wild-type flies. Also, Lee and Edery [33] showed that *Drosophila*'s ability to fight infections is under circadian control and that flies are significantly more resistant to bacterium when infected in the middle of the night than during the day.

It has been shown that several genes from *Aedes aegypti* are up or down-regulated upon Dengue virus infection, and in DENV-2 infected mosquitoes at least one orthologue (AAEL012562) of a *Drosophila* gene involved in the control of circadian rhythms, *Clock*, has its expression nearly doubled after infection [29]. We believe this variation in a gene probably central to the control of mosquito circadian rhythms could also contribute to the observed changes in activity behavior and we are currently investigating whether Dengue virus infection alters the circadian expression patterns of other clock genes [21].

We are aware that our study suffers from possible caveats. For example, we see a large variation in behavioral effects of Dengue infection between experiments that we cannot explain at the moment. Nevertheless, our study shows that Dengue infection increases mosquito locomotor activity. Changes in vector behavior caused by infection can have potential epidemiological implications. Our results encourage further studies to assess whether increased locomotor activity could have an impact on virus transmission dynamics and Dengue epidemiology.
